# Necrotizing Skin Findings in Coronavirus Disease 2019: A Case Report

**DOI:** 10.30699/ijp.2020.128904.2408

**Published:** 2020-10-10

**Authors:** Fatmagül Kuşku Çabuk, Damlanur Sakiz

**Affiliations:** 1 Dr Sadi Konuk Training and Research Hospital, Department of Pathology, Istanbul, Turkey

**Keywords:** Acroischemia, COVID 19, Microthrombosis, Necrosis, Vasculitis

## Abstract

Coronavirus is a single-stranded RNA virus that causes acute respiratory syndrome and various skin lesions. In addition, ischemic acral lesions have been reported in patients with severe coronavirus disease 2019 (COVID-19) due to coagulation disorders. We here present a case with ischemic acral lesions caused by COVID-19. The patient was 51-year old male who was hospitalized with COVID-19 pneumonia. After 28 days in the Intensive Care Unit, patient developed acroischemic lesions in the fingers and toes. In the histopathologic examination, vasculitis was observed as the infiltration of mixed-type inflammatory cells in the mid-sized muscular arteries wall. Moreover, microthrombosis was detected in small capillaries. It is clear that thrombotic lesions have occurred as a result of COVID-19 or administered treatment. Further studies are required to confirm and better characterize the skin reactions in COVID-19.

## Introduction

Coronavirus disease 2019 (COVID-19) emerged in Wuhan, China in December 2019 and resulted in a pandemic spread all over the world. Coronavirus is a single-stranded RNA virus that causes severe acute respiratory syndrome (1). The COVID-19 enters human cells by attaching angiotensin-converting enzyme 2 (ACE2), which is highly expressed in lung alveolar cells, cardiac myocytes, vascular endothelium, and some other cells (2). 

This disease often represents with high fever, cough, headache, dyspnoea, anosmia, and weakness. Less frequently rhinorrhoea, diarrhea, and myalgia are found and skin lesions are rarely reported (3). Patients especially develop pneumonia that causes damage to alveolar epithelial cells, hyaline membrane formation, type II pneumocytes hyperplasia, diffuse alveolar damage, and fibrosis. Moreover, secondary bacterial infections might be added to the table in later stages (4). Cardiovascular complications can be observed in patients, even in case of no previous cardiovascular disease. The cardiovascular signs can range from hypoxia to severely disseminated intravascular coagulation (DIC) (2).

Although disease prognosis is poor in older patients and cases with comorbidity, such as cardiovascular disease, young and healthy patients are also at risk for complications (4). Different skin lesions are rarely reported in COVID-19, including urticaria, maculopapular eruption, vesicular eruption, morbilliform eruption, chickenpox-like, and erythema multiforme-like purpuric eruption (5-11). Patients refer with the complaints of burning, pain, and chill burns (8). Ischemic acral lesions have been reported to occur in patients with severe COVID-19 due to coagulation disorders.

##  Case Report

A 51-year-old male patient referred to Dr. Sadi Konuk Training and Research Hospital, İstanbul, Turkey at the end of March 2020 with the complaint of weakness and dry cough for 11 days. At the time of admission his signs were as follow: fever 36.2°C, pulse 97/min, oxygen saturation 92%, CRP 118.97 mg/L (ref: < 5 mg/L). The patient was hospitalized and treatment was started because of low oxygen saturation. He had only comorbidity with chronic hepatitis B (HBsAg 100). There was obvious bilateral infiltration in the lungs on thorax computerized tomography. The result of the polymerase chain reaction (PCR) for COVİD-19 using nasopharyngeal swab sample was positive. 

In the follow-up, he was taken to the Intensive Care Unit (ICU) because of a decrease in oxygen concentration. On the 13^th^ day of intensive care therapy, myoglobulin level, proBNP, troponin I, fibrinogen, and D-dimer were detected as 1029 ng/mL (<10 ng/mL), 8660 ng/l (0-133), 88 pg/mL (0-17.5), 500 mg/dl (200-400), and 2.87 µgFEU/mL (0-0.5), respectively. There were mild elevations in prothrombin time, international normalized ratio (INR), and fibrinogen level with the values of 16.3 sec (11-15), 1.25 (0.8-1.2), and 412 mg/dL (200-400), respectively. The prothrombin level was found to be slightly lower than normal 152 10^3^/uL (155-366). 

**Fig. 1:**
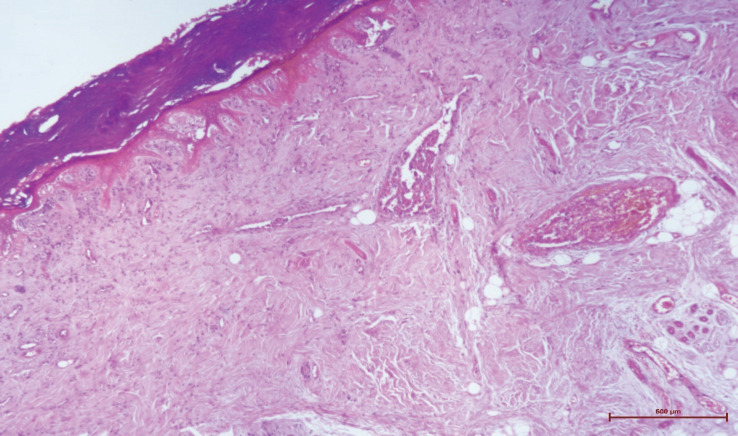
Necrosis in the epidermis (haematoxylin eosin x40)

**Fig. 2:**
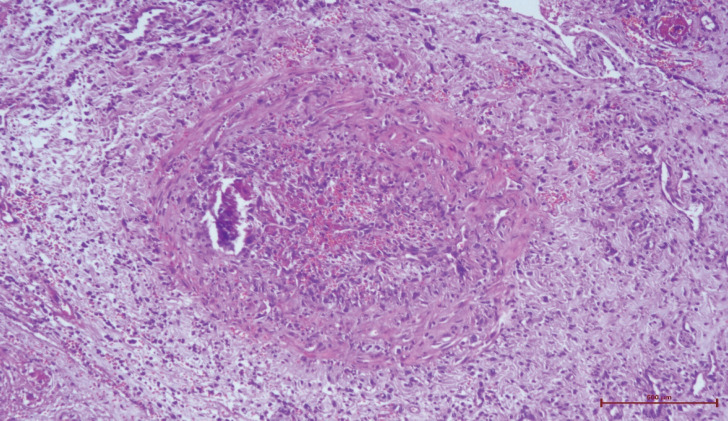
Thrombus formation in the medium-sized vascular structure (haematoxylin eosin x40)

**Fig. 3:**
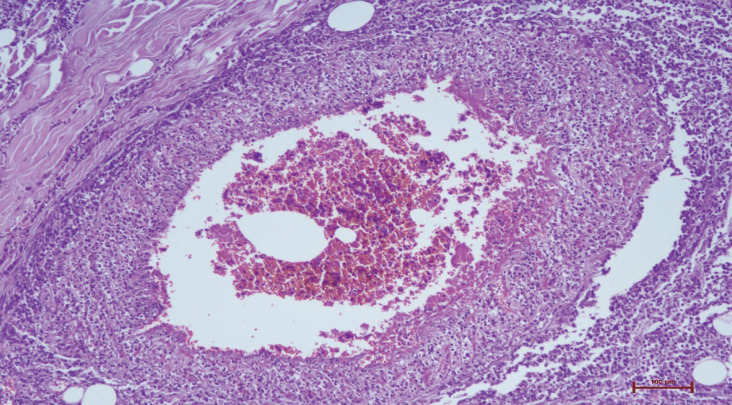
Neutrophil leukocytes accumulation in the lumen in the medium-sized diameter vascular wall (haematoxylin eosin x200)

**Fig. 4:**
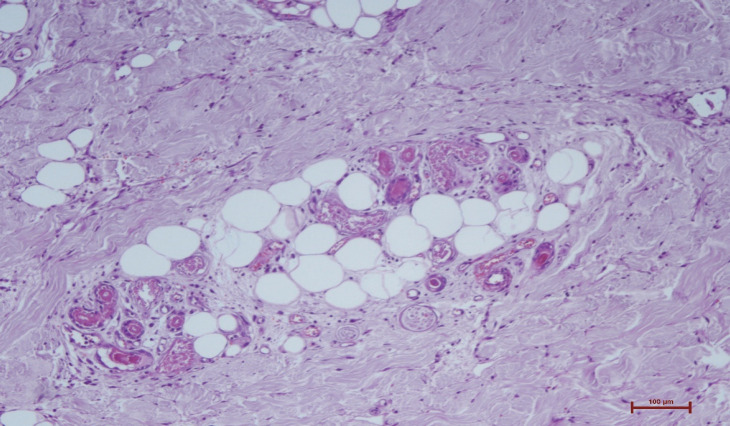
Microthrombus formation in small vascular structures (haematoxylin eosin x200)

After 28 days, necrosis developed in the left hand and left toes. Necrosis was detected in the distal ends of the 1^st^, 2^nd^, and 3^rd^ fingers of the left hand and the 1^st^, 2^nd^, and 4^th^ left toes leading to amputation. In the histopathologic evaluation, necrosis in the epidermis, dermis, and subcutaneous tissue were noted (Figure 1). Severe acute inflammation in the focal area of the subcutaneous adipose tissue and vasculitis with mixed-type inflammatory cells infiltration in the mid-sized muscular arteries wall were observed (Figures 2, 3). In addition, microthrombosis was detected in small capillaries (Figure 4). The patient is still being treated in the ICU.

## Discussion

Acroischemic skin lesions were noted in an asymptomatic child 5 weeks after the first case occurred in Italy. These lesions are mostly found in healthy children and adolescents and develop often in toes, sometimes in fingers, and rarely in plantar areas. Whole fingers and toes are not affected and the lesion areas are separated by a sharp demarcation line. These skin lesions emerge as painful, reddish-purple, or bluish bullous or represent as blackish crusts that may rotate and heal after about 2 weeks (9). 

The acute self-healing acroischemic lesions are different from other chronic conditions, such as acrocyanosis, perniosis, Henoch-Schönlein type vasculitis, and are not as severe as meningococcal sepsis or protein C deficiency. However, these may be due to secondary microthrombosis resulting from endothelial damage and vascular disorders (9). Skin lesions are mostly found as urticaria, erythema multiforme-like targetoid, red-purple plaque macular lesions, and rarely as nodular lesions (6, 8). These lesions can be rarely observed as ischemic, red to violet plaques, macules, and nodules on the distal parts of fingers and toes (6). 

Moreover, there are reports of the signs of vasculitis frequently found in the pediatric and adolescent age groups with negative PCR results for COVID-19 (12, 13). The mean age of the patients with skin lesions was reported to be 19.9 years with a range of 1-51 years. However, there are reports similar to our patient that skin lesions occurred in older ages of 58-77 years. It should be noted that the necrosis of extremities was not observed in these patients (10). 

The COVID-19 symptoms started before skin lesions in these patients. It is stated that skin symptoms may occur approximately 8.7 days (2-24 days) after COVID-19 symptoms (6). It was reported that cutaneous findings disappear in an average of 48 h (24 h to 6 days) and most of the skin lesions appear in young asymptomatic patients (8). 

However, in our case, skin lesions appeared nearly one month following admission in the ICU with severe pneumonia symptoms. Skin lesions occurred as necrotic red-purple ischemic lesions. Histologic examination revealed signs of vasculitis infiltrated by mixed-type inflammatory cells in the medium-sized vessels. The mentioned findings are similar to the vasculitis found in polyarteritis nodosa (PAN) (14). 

Our patient was affected by chronic hepatitis B. Although PAN may develop in hepatitis B patients, microthrombosis in small vascular structures observed in the present case is not an expected finding in PAN (14). Furthermore, other organs, such as the kidney might be involved in PAN. Nonetheless, in our patient, there was no laboratory finding concerning the involvement of other organs. As a result, we can conclude that histopathologic changes are due to COVID-19. 

In the study performed by Xiaohong *et al.*, the thickening of the alveolar wall in the lung, inflammatory infiltrate in the alveolar wall, congestion in the capillaries, and thrombi in the vascular structures were observed in autopsy cases (15). In addition, hypertrophy, degeneration, necrosis, and inflammation of mixed-type cells were reported in the heart. Similar findings were detected in other organs, including the liver, kidney glomeruli, pancreas, and stomach. Mild lymphocytic inflammation around the superficial blood vessels in the dermis was observed in the skin lesions (16). 

Cordoro *et al.* detected lymphocytic vasculitis without thrombosis in small dermal vessels. All these patients were at the adolescent age and their COVID-19 test was found as negative (12). Acute respiratory distress syndrome provides the potential for hypoxic pulmonary vasoconstriction, pulmonary hypertension, and right ventricular failure in patients with COVID-19. In our patient, heart failure developed and was confirmed by increased blood proBNP. At the same time, troponin and myoglobin levels showing myocardial damage augmented in our case. In COVID-19, elevated cardiac troponin levels are associated with poor prognosis (2). 

Necrosis in acral areas in patients with COVID-19 has been reported to be associated with DIC in severe cases (6). In these patients, D-Dimer, prothrombin, and fibrinogen levels rise, which is accompanied by poor prognosis (2, 15). In the present case, D-dimer, fibrinogen concentration, and INR were high and bleeding time was slightly prolonged but our patient is still alive after three months. However, it is not yet known whether these hemostatic alterations are specific effects of COVID-19 or are the result of a cytokine storm that accelerates the onset of systemic inflammatory response syndrome as observed in other viral diseases. 

Severe inflammatory response, critical illness, and underlying risk factors may predispose to thrombotic events similar to previous zoonotic coronavirus outbreaks. Treatments for COVID-19 may result in a predisposition to thromboembolism as they may have negative drug interactions with antiplatelet agents and anticoagulants (2).

The only report of the relationship between this type of lesions and COVID-19 in the literature describes the cyanosis and gangrene of the toe in severe COVID-19 cases (9). Our COVID-19 positive patient with chronic hepatitis B had necrosis and PAN-like vasculitis findings on the fingers and toes.

## Conclusion

The COVID-19 has a different course in patients with chronic diseases. Therefore, more data is required to learn how this disease interacts with thrombotic disorders (2). It is not understood whether these depend on COVID-19 or are the sequels of administered treatments. Further studies are needed to confirm and better characterize the skin reactions in COVID-19.
